# Immunomodulatory molecule PD-L1 is expressed on malignant plasma cells and myeloma-propagating pre-plasma cells in the bone marrow of multiple myeloma patients

**DOI:** 10.1038/bcj.2015.7

**Published:** 2015-03-06

**Authors:** S Yousef, J Marvin, M Steinbach, A Langemo, T Kovacsovics, M Binder, N Kröger, T Luetkens, D Atanackovic

**Affiliations:** 1 Division of Hematology and Hematologic Malignancies, University of Utah, Huntsman Cancer Institute, Salt Lake City, UT, USA; 2 Department of Stem Cell Transplantation, University Medical Center Hamburg-Eppendorf, Hamburg, Germany; 3 Flow Cytometry Core Facility, University of Utah Health Sciences Center, Salt Lake City, UT, USA; 4 Department of Internal Medicine II; Oncology/Hematology/Bone Marrow Transplantation with the section Pneumology, University Medical Center Hamburg-Eppendorf, Hamburg, Germany

Multiple myeloma (MM) is the second most common hematological malignancy and develops from a malignant plasma cell (PC) clone within the human bone marrow (BM). The proliferation and expansion of the malignant PC producing a monoclonal immunoglobulin eventually results in BM failure with anemia, skeletal involvement with osteolytic lesions and hypercalcemia, and renal failure due to the excessive production of paraprotein.^1^

There have been significant therapeutic advances over the past decade and the median survival of myeloma patients has increased to ~6 years.^2^ However, most patients will still eventually suffer a fatal relapse after an initial effective therapy. This is due to the persistence of chemotherapy-resistant precursor cells^3^ in the BM even after destruction of the bulk of tumor cells^4^ and, accordingly, the disease will become more and more refractory to chemotherapy after each additional line of treatment.

Immunotherapy could play an important role in eradicating chemotherapy-resistant myeloma cells from the BM of the patients and one approach in particular has been shown to be very promising. Monoclonal antibodies targeting PD-L1 or its receptor PD-1, which is expressed on the surface of exhausted T and B cells and is involved in the maintenance of peripheral self-tolerance,^5^ have shown significant clinical efficacy in different solid tumors^6, 7^ and also hematological malignancies.^8^ PD-L1 is expressed on different antigen-presenting cells and it has been suggested that PD-L1 is absent from normal PCs^9, 10^ while it is expressed on myeloma cell lines^9^ and primary myeloma tumor cells from patients with MM.^9, 10^ However, so far it has remained controversial whether normal PCs from myeloma patients or healthy donors are also PD-L1 positive and it is unclear whether quiescent myeloma-propagating cells express PD-L1 as a therapeutic target.

We recently performed a flow cytometry-based analysis of BM samples from 11 healthy donors, 15 patients with monoclonal gammopathy of undetermined significance, 6 patients with smoldering MM and 51 patients with MM. All the patients and healthy subjects had identifiable CD38+/CD138+ PCs in their BM but, as expected, the number of PCs was the highest in our patients with active MM (Figure 1a). When we determined the percentage of PD-L1-expressing PC in each subgroup, we found that in all subjects the vast majority of cells were PD-L1 positive, however, PD-L1 expression was somewhat more commonly observed on the tumor cells of patients with PC dyscrasias than on the PCs from healthy donors (Figure 1b).

Next, we determined the expression level of PD-L1 on phenotypically aberrant PC within the BM of patients with all three types of PC dyscrasias (*n*=22) as defined by the absence or presence of CD19/CD56 and CD45/CD28, respectively. We did not detect any difference in the expression of PD-L1 among the patients' PC subtype as defined by positivity for CD19 and CD56, in particular we did not observe an abnormal expression on those PCs showing the typical CD19−/CD56+ aberrant phenotype (Figure 1c). However, we observed a significantly increased expression of PD-L1 on those PCs showing the aberrant CD45+/CD28+ phenotype versus, for example, normal CD45+/CD28− PC (Figure 1d).

It has recently been shown that myeloma-propagating activity is the exclusive property of a cell subpopulation characterized by its ability for bidirectional transition between the dominant CD19−CD138+ PC fraction and a low frequency fraction of CD19−CD138− pre-PCs.^3^ Importantly, it has been demonstrated that pre-PCs are more quiescent, are enriched in epigenetic regulators and are up to 300-fold more drug resistant than the common malignant PCs of myeloma patients.^3^ Using multicolor flow cytometry, we next analyzed the expression of PD-L1 on both fractions of myeloma-propagating cells in the BM of our patients (Figure 2a). We found that in all myeloma patients analyzed, conventional CD138-positive PC as well as CD138-negative pre-PC myeloma-propagating cells expressed similar high levels of surface molecule PD-L1 (Figure 2b).

In conclusion, we have shown here in a large collection of patients with PC dyscrasias including MM that the immunomodulatory molecule PD-L1 is commonly and broadly expressed on the patients' PCs. This and our observation that PD-L1 shows an increased expression on those myeloma cells with an aberrant phenotype suggests an important function of this molecule in suppressing local immunity in the tumor microenvironment of MM. Indeed, it has been shown that myeloma cells overexpressing PD-L1 inhibit the generation of cytotoxic T cells *in vitro*, an immunosuppressive effect which can be prevented by pre-incubation with an anti-PD-L1 antibody.^9, 11^ In addition, Feyler *et al.*^12^ demonstrated that co-culture of CD4^+^ T cells with myeloma cells resulted in the generation of CD4^+^CD25^+^FoxP3^+^ T-regulatory cells (Tregs) in a contact-dependent manner. These Tregs demonstrated a suppressive phenotype and an increased PD1 expression compared with naturally occurring Tregs.^12^ Furthermore, It has recently been shown that the PD-1/PD-L1 pathway not only promotes progression of myeloma indirectly by causing failure of immune control but that BM stromal cells induce expression of PD-L1 on myeloma cells, which results in increased proliferation of the tumor cells and dampened susceptibility to anti-myeloma chemotherapy. Accordingly, clinical progression was observed in patients whose myeloma cells expressed high levels of PD-L1.^13^

These combined data and the observation made by others that the expression of the PD-L1 receptor PD-1 is upregulated on T cells isolated from patients with MM,^14^ would support the initiation of clinical trials investigating the efficacy of anti-PD-1 or -PD-L1 antibody treatment in MM either alone or in combination, for example, with lenalidomide.^15^ In addition to indirectly targeting MM by reversing local immunosuppression through anti-PD-1/PD-L1 antibody treatment one could also imagine to directly target PD-L1 expressed on the myeloma cells, for example, by the adoptive transfer of PD-L1-specific chimeric antigen receptor-transduced T cells. As we have shown here, PD-L1 is also expressed on myeloma-propagating pre-PCs and, accordingly, immunotherapeutic approaches directly targeting this surface molecule would have the potential to eradicate chemotherapy-resistant disease. It remains to be determined whether the expression of PD-L1 on normal PCs, as demonstrated in our current study, represents an obstacle to approaches like this.

## Author contributions

SY performed the research, performed the statistical analysis, analyzed and interpreted the data, and wrote the manuscript; JM performed the research; MS collected the data; AL collected the data; TK collected the data; MB performed the research; NK collected the data and contributed vital new reagents or analytical tools; TL wrote the manuscript, DA designed the research, performed the statistical analysis, analyzed and interpreted the data, and wrote the manuscript.

## Figures and Tables

**Figure 1:**
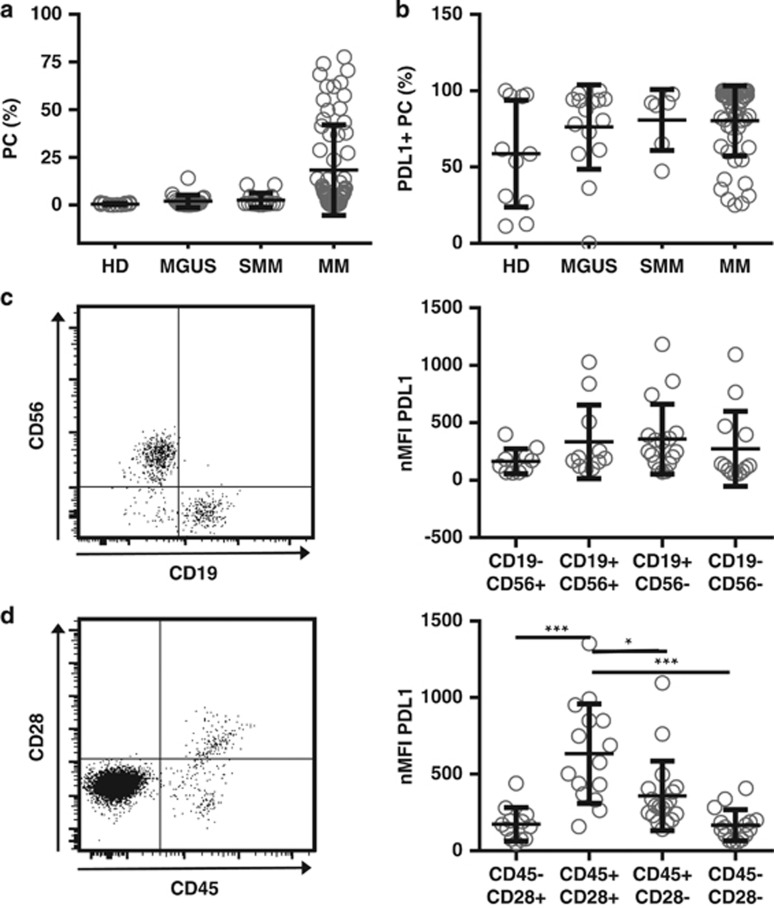
PD-L1 is expressed on malignant and normal plasma cells. (**a**) Bone marrow of healthy donors (HD, *n*=11), monoclonal gammopathy of undetermined significance (monoclonal gammopathy of undetermined significance (MGUS), *n*=15), smoldering myeloma (SMM, *n*=6), and multiple myeloma patients (MM, *n*=51) were analyzed by flow cytometry for percentages of plasma cells by gating on CD38+CD138+ cells after doublet exclusion. (**b**) Percentages of PD-L1 positivity were assessed in relation to matched isotype or FMO controls, using a mouse anti-human PD-L1 monoclonal antibody (clone 29E.2A3; BioLegend, San Diego, CA, USA). Only those samples with event numbers >100 within the PC gate were taken into account. (**c**) Normalized (subtraction of FMO control) median fluorescence intensity (nMFI) of PD-L1 was assessed on different PC subsets of SMM, MGUS and MM patients (*n*=22) according to their CD56 or CD19 expression or (**d**) CD28 and CD45 expression (exemplary dot blots on the left). Only those samples where clear populations were detectable and the number of events was >50 were taken into account. All data are presented as mean±s.d. Statistical significance was evaluated using analysis of variance followed by Tukey's multiple comparison test. *P<0.05, ***P<0.001.

**Figure 2:**
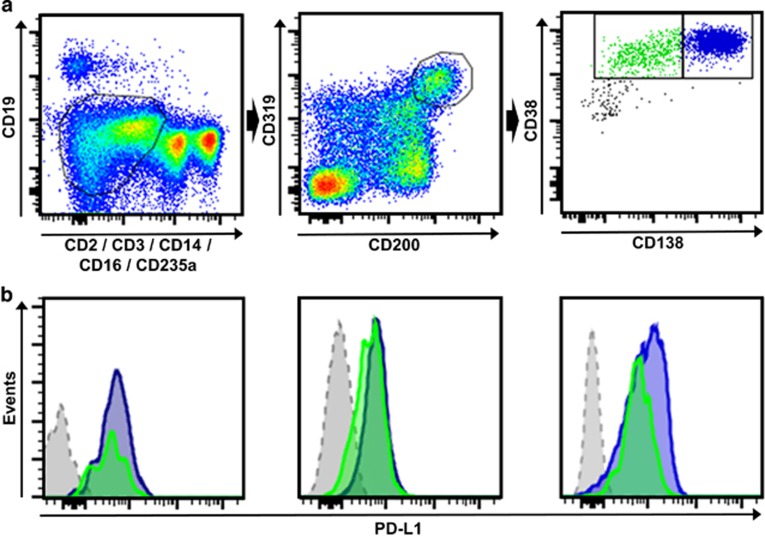
PD-L1 is expressed on myeloma-propagating cells and the pre-PC subpopulation. (**a**) An exemplary gating scheme for myeloma-propagating cells is shown. After doublet exclusion, the gate was set on CD19−, CD2−, CD3−, CD14−, CD16−, CD235a− (left) and cells were then gated for CD200+CD319+ (middle). Myeloma-propagating cells (right) were differentiated into CD38+CD138^high^ (blue, PC) and CD38+CD138^low/negative^ (green, pre-PCs) as previously described.^3^ (**b**) Histograms show MFI of PD-L1 in three different MM patients. The blue histogram represents CD38+CD138^high^ PC and the green histogram shows CD38+CD138^low/negative^ pre-PCs. The gray histogram represents the FMO control gated on CD319+CD200+ cells.
